# Oxidative Stress: A New Pathophysiological Pathway in Parkinson's Disease and a Potential Target of the Brain-Sport Crosstalk

**DOI:** 10.1155/padi/6691390

**Published:** 2025-03-21

**Authors:** Stefano Caproni, Alessio Di Fonzo, Carlo Colosimo

**Affiliations:** ^1^Neurology Division and Stroke Unit, Neuroscience Department, Santa Maria University Hospital, Terni, Italy; ^2^Neurology Unit, Neuroscience and Mental Health Department, IRCCS Ca' Granda Ospedale Maggiore Policlinico Hospital, Milan, Italy

**Keywords:** dopamine, irisin, mitochondria, oxidative stress, Parkinson's disease

## Abstract

Oxidative stress (OS), a condition that occurs when the balance between reactive oxygen species production and antioxidant defense mechanisms is disrupted, has been implicated in the pathogenesis of several neurological conditions, including neurodegenerative and vascular disorders. Ferroptosis is a mechanism mediating OS-induced damage, with growing evidence of specific involvement in both Parkinson's disease (PD) and ischemic stroke. Regular physical activity may have an antioxidant effect by increasing the production and activity of nonenzymatic and enzymatic antioxidants. Among the biological mediators of physical activity, irisin may act as an agent capable of inducing systemic changes and crossing the brain-blood barrier. This review aims to describe the main role of OS in the pathophysiology of PD, highlighting putative neurodegenerative mechanisms and emphasizing the potential targeting by physical activity as a possible shared preventive and symptomatic treatment approach.

## 1. Introduction

Parkinson's disease (PD) is one of the most common neurological diseases in the general population, particularly in people over 60 years of age of both sexes. It represents a pivotal neurological cause of disability by impacting considerably on patients' quality of life [1]. Hence, PD is the subject of increasing interest among clinicians, with considerable efforts being made to better understand their pathophysiology and thus optimize their treatment [2].

Beyond neurodegenerative mechanisms, it has been largely observed that vascular risk factors can play a role in PD pathophysiology [3]. The relevance of vascular risk factors in degenerative and secondary movement disorders has also been confirmed by several neuroimaging studies [4]. From the opposite point of view, protective behaviors toward the vascular risk factor, as well as regular physical activity (PA), appeared to exhibit an inverse correlation with PD [5]. This phenomenon can be explained by the inhibition of dopaminergic neuron loss within the substantia nigra (SN) and basal ganglia, which play a crucial role in motor coordination and regulation [6]. Such adaptive mechanisms involve the modulation of dopamine (DA) and glutamate (GLU) neurotransmission [7].

**Figure 1:**
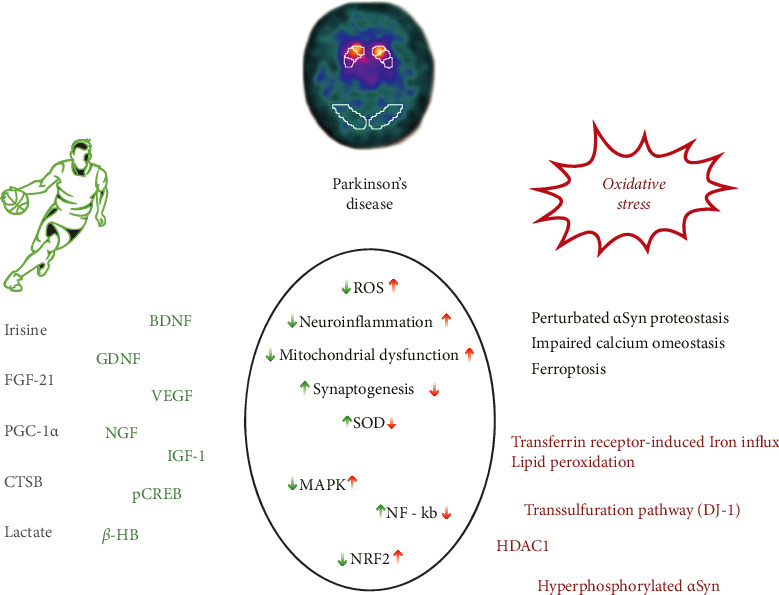
Main pathophysiological mechanisms of Parkinson's disease targeting oxidative stress-induced damage and the protective effects of physical activity. ADMA, asymmetric dimethylarginine; BDNF, brain-derived neurotrophic factor; *β*-HB, *β*-hydroxybutyrate; CTSB, cathepsin B; FGF-21, fibroblast growth factor 21; GDNF, glial-derived neurotrophic factor; GPX4, glutathione peroxidase 4; HDAC1, histone deacetylase 1; IGF-1, insulin-like growth factor-1; MAPK, mitogen-activated protein kinase; MMP, matrix metalloproteinase; NF-kB, nuclear transcription factor-kappa B; NGF, nerve growth factor; Nrf2, nuclear factor erythroid two p45 subunit-related factor 2; NO, nitric oxide; pCREB, phosphorylated cAMP response-element binding protein; PGC-1α, some-proliferator-activated receptor *γ* coactivator-1α; ROS, reactive oxygen species; SOD, superoxide dismutase; VEGF, vascular endothelial growth factor.

Here, we aim to review the main role played by oxidative stress (OS) in the pathophysiology of PD, highlighting the common neurodegenerative mechanisms and emphasizing the potential targeting by PA, as a possible preventive and treatment approach (Figure 1).

## 2. OS and PD: Pathophysiological Mechanisms

OS is considered to play a pivotal role in PD pathophysiology, as it is counted, together with energy stress and altered proteostasis, as the most relevant pathogenetic process involved in the progression of PD [8]. OS is strictly linked to mitochondrial dysfunction, leading to intracellular reactive oxygen species (ROS) accumulation, intracellular calcium influx with caspase activation, perturbated *α*-synuclein (αSyn) proteostasis, and altered ubiquitin-proteasome system. Specifically, the accumulation of αSyn in mitochondria is supposed to cause mitochondrial complex I deficits in [9, 10].

The function of mitochondrial complex I, an essential component of the electron transport chain, is compromised in various tissues obtained from individuals with PD. Peroxisome proliferator-activated receptor-*γ* (PPARγ) co-activator 1α (PGC1α), a pivotal regulator of mitochondrial transcription, exhibits reduced activity in PD, resulting in the under-expression of its target genes. According to current theories, mitochondria typically contain low levels of αSyn. However, when αSyn accumulates within mitochondria, it leads to impairments in mitochondrial complex I and triggers OS [11].

An additional link between ROS and PD is emerging from the study of early-onset monogenic forms associated with loss of function mutations of the PINK1 and PRKN genes. These genes encode for two essential proteins that act as sensors of mitochondrial membrane depolarization and ubiquitination. Thus, the finding of loss of function mutations in PD patients supports a role, in the PD pathology, of defective mitophagy, a mechanism ensuring mitochondrial quality via the clearance of damaged mitochondria by autophagy and lysosomal degradation [12]. OS emerged as a key pathogenic factor also in models of defective DJ-1 and LRRK2 proteins, which are encoded by other PD-associated genes [13].

A growing investigation of how familial PD genes, such as LRRK2 mutations, disrupt mitophagy and the recognition that αSyn oligomers and aggregates interact with substances on the outer mitochondrial membrane, leading to mitochondrial dysfunction, could shed light on the still poorly understood molecular mechanisms underlying PD [14].

Other links between OS and PD reside in the peculiar characteristics of dopaminergic neurons of SN pars compacta, which are the cells primarily damaged in PD. For example, a possible pathogenic role could be exerted by the monoamine oxidase B (MAO-B), the enzyme that degrades DA. MAO-B promotes the GLU accumulation and the release of free radicals, causing excitotoxicity [15].

In the context of PD development, ROS also influences the stability of nucleic acids, leading to RNA oxidation and mitochondrial DNA mutations. Concurrently, αSyn, parkin, and proteasome proteins in the central nervous system (CNS) undergo oxidative modifications, resulting in their increased aggregation and instigating an inflammatory response. Thus, there is an observed indirect correlation between the toxicity of the phosphorylated αSyn protein and the elevated levels of ROS or nitrates, indicating the prominence of Lewy bodies, typically found in CNS of individuals with PD [16].

Grofik et al. analyzed blood OS parameters in 125 PD patients and 55 healthy controls, pointing out that uric acid seems to be associated with PD, while glutathione (GSH) and bilirubin were associated with PD only in female patients, even in the absence of predictive or diagnostic power [17].

Astrocities and microglia are an additional target of OS implicated in neurodegenerative processes. Glial cells contribute to compensatory counteracting the OS due to excessive DA firing in a specific nigral subpopulation. Intriguingly, both astrocytes and microglia possess DA receptors, which become more active in the context of neuroinflammation. The stimulation of DA receptors has garnered attention due to its anti-inflammatory effects, particularly because the functions of astrocytes and microglia are significantly impacted by both the depletion of DA and the therapeutic replacement of DA in PD [18].

## 3. Ferroptosis: A Risk Factor of PD

Ferroptosis is a recently discovered form of regulated cell death that depends on iron and exhibits distinct morphological and biochemical characteristics compared to other programmed cell death modes such as apoptosis and necroptosis [19]. Morphological changes in ferroptosis include shrunken mitochondria with a disrupted outer membrane, reduced crista, a compressed inner membrane, and an intact cell nucleus. In contrast, apoptosis and necroptosis typically involve swollen mitochondria and fragmented nuclei [20]. Biochemically, ferroptosis is characterized by iron overload and the toxic accumulation of lipid peroxides and ROS within cells, leading to the generation of numerous alkyl-oxygen radicals that cause severe damage and disorganization of the cell membrane [21].

Since iron turnover is slow and there is no physiological mechanism for its excretion, iron can accumulate in the brain and contribute to age-related pathophysiology in diseases such as PD through OS-induced damage. Notably, ferritin, a protein involved in iron storage, has been implicated in neurodegenerative processes [22].

Ferroptosis exhibits various similarities with the pathophysiology of PD. Particularly, αSyn has functional connections with both iron and lipid metabolism, suggesting a potential interplay between dysregulated αSyn and other PD-associated pathological features related to ferroptosis [23]. Several key features and triggers of the ferroptotic cell death pathway, such as iron overload, elevated lipid peroxidation, and reduced levels of GSH, are also prominent in PD pathological processes. Considering these shared characteristics, it is plausible to hypothesize a strong involvement of ferroptosis in PD neurodegeneration [24].

Studies have demonstrated the prevalence of ferroptosis as a form of cell death in various in vitro, ex vivo, and in vivo models of PD. Iron accumulation is particularly prominent in brain regions associated with neurodegenerative disorders. In PD, increased iron levels are specifically observed in glial cells and dopaminergic neurons of the SN pars compacta, and these levels correlate with disease severity [25]. Dysregulated modulation of iron import and efflux likely contributes to intracellular iron elevation, leading to heightened vulnerability to free radical formation and ferroptosis. Given the extensive evidence supporting the impact of iron on PD pathology, iron chelation has been explored as a potential therapeutic strategy in various models of PD that induce an OS response resembling the PD phenotype [26].

In PD patients, dysfunction of Complex I is present in the SN, frontal cortex, fibroblasts, and platelets, resulting in increased superoxide production. Complex I deficiency is associated with a deficit in coenzyme Q10 (CoQ10), further contributing to ROS production in mitochondria and lipid peroxidation in membranes [27]. Interestingly, recent findings indicate that CoQ10 plays a significant antiferroptosis role through an axis involving FSP1 (ferroptosis suppressor protein 1) and NADPH-CoQ10 [28]. A study by Sian et al. investigated GSH levels in postmortem brain regions from patients with PD, progressive supranuclear palsy, multiple system atrophy, and Huntington's disease, revealing a specific 40% reduction in GSH levels only in the substantia nigra of PD patients [29].

Recently, Cao et al. have shown that DJ-1 acts as a ferroptosis inhibitor by preserving the transsulfuration pathway and thereby the biosynthesis of cysteine and GSH. DJ-1 depletion leads to lipid ROS accumulation and a heightened sensitivity to ferroptosis cell death. The discovery of DJ-1 as a ferroptosis suppressor further supports that ferroptosis is implicated in PD pathology [30].

The combination of elevated iron levels and high concentrations of polyunsaturated fatty acids (PUFAs) within dopaminergic neurons creates a highly vulnerable environment to lipid peroxidation, making even slight imbalances in iron, DA, or lipid homeostasis capable of sensitizing these neurons to ferroptosis. Understanding the distinct and regulated pathways of lipid peroxidation is crucial for deciphering the underlying neuropathology involved in the death of nigral cells in PD and for developing therapeutic strategies aimed at inhibiting ferroptosis [31].

Several studies have highlighted the protective role of Keap1/Nuclear factor erythroid-2-related factor 2 (Nrf2), a master regulator of the antioxidant response, against ferroptosis. In PD patients, Nrf2 and its downstream effectors are found to be highly transcribed in blood leukocytes compared to controls. Notably, the levels of Nrf2 transcripts correlate with the duration of PD, suggesting that Nrf2 is involved in combating the intrinsic OS observed in the disease pathology [32]. Moreover, in the SN of PD patients, Nrf2 is predominantly localized in the nuclei. This nuclear translocation of Nrf2 in PD indicates that dopaminergic neurons are inherently vulnerable to OS, potentially leading to the recruitment of Nrf2 as a cellular defense mechanism [33]. Additionally, αSyn oligomers interacting with iron in neurons induce the production of ROS and lipid peroxidation, while reducing GSH levels, ultimately leading to ferroptosis via iron-dependent oxidation. This finding is particularly significant, given that dopaminergic neurons, which are susceptible to PD, exhibit high levels of iron and inherently reside in an oxidative environment due to their DA metabolism [24]. Iron chelation has been demonstrated to provide neuroprotection against neurotoxin insults related to PD, reduce αSyn aggregation in vitro, and rescue behavioral deficits induced by iron exposure in a mouse model of αSyn aggregation. Collectively, studies on iron and PUFA dependence suggest that over time, the physiological and/or pathological functions of αSyn may create a proferroptotic environment in dopaminergic neurons [34].

## 4. Physical Activity and Brain Health: Biochemical and Physiological Principles

The susceptibility of the brain to energy deficits, OS, and disrupted cell death signaling becomes more evident as age progresses, leading to increased mitochondrial dysfunction, oxidative damage, and impaired clearance of cellular waste. Although the beneficial effects of exercise-induced mitochondrial adaptations may be diminished with advancing age, their positive impact remains throughout life [35].

Exercise-induced transient elevation of ROS levels can influence redox regulation not only in skeletal muscles but also in the brain. These ROS fluctuations stimulate adaptive responses in the brain, similar to skeletal muscles, resulting in enhanced endogenous antioxidant defenses. Regular exercise is likely to provide neuroprotection against neurodegenerative diseases by upregulating antioxidant stress defenses, promoting beneficial mitochondrial adaptations, and indirectly regulating neuroprotective factors such as brain-derived neurotrophic factor (BDNF) through ROS signaling [36].

Irisin, a myokine released during exercise, is derived from the cleavage of the transmembrane precursor protein fibronectin type III domain-containing protein 5 (FNDC5), which is expressed in muscles under the control of PGC-1α. Aerobic exercise serves as a potent stimulus for irisin secretion. The effects of irisin include the disinhibition of BDNF through histone deacetylase HDAC-mediated inhibition in the brain [37]. The physiological benefits of irisin are evidenced by its positive correlation with skeletal muscle mass and aerobic capacity. Moreover, both acute and chronic exercise lead to increased irisin release into the bloodstream. Intriguingly, irisin has been detected in brain tissue and is associated with neuronal differentiation in mouse embryonic stem cells. It is believed that irisin mediates the beneficial effects of PA by enhancing synaptic function through the upregulation of BDNF [38].

Regular exercise yields clear advantages for brain health, including molecular changes such as decreased ROS production and oxidative damage, improved enzymatic antioxidant defenses, and notable effects on components involved in mitochondrial biogenesis and adult neurogenesis in the hippocampus [12].

Ketone bodies, which are water-soluble molecules derived from fatty acids and synthesized in the liver, are released into the bloodstream during physical exercise, particularly *β*-hydroxybutyrate (*β*-HB) [39]. *β*-HB can enter the brain through monocarboxylate transporters and has previously been recognized for its neuroprotective effects in PD. It can cross the blood brain barrier (BBB) and enhance BDNF transcription by activating BDNF promoters. Direct administration of *β*-HB to neurons in vitro has been shown to increase BDNF expression. Furthermore, exercise prompts the liver to release fibroblast growth factor 21 (FGF21), a hepatokine [40]. In obese, insulin-resistant rats, subcutaneous injection of FGF21 prevented cognitive decline by improving hippocampal synaptic plasticity, dendritic spine density, and brain mitochondrial function and reducing cell apoptosis [37].

The relationship between PA and OS is highly complex and depends on various factors such as the mode, intensity, and duration of exercise. Regular moderate training appears to be beneficial for OS and overall health. On the other hand, acute exercise can lead to increased OS, although this stimulus is necessary to trigger an upregulation of endogenous antioxidant defenses (hormesis) [41].

The brain indirectly senses the effects of PA through adipose tissue (adiponectin) or the liver (FGF-21 and IGF-1). Myokines facilitate communication between muscles and various organs such as the liver, gut, pancreas, adipose tissue, bone, vascular system, skin, and brain. Cathepsin B, for instance, can enhance the expression of BDNF mRNA and increase BDNF protein levels, as well as stimulate the production of doublecortin, a protein known for its neuroprotective effects by promoting neuronal migration [42]. Exercise may also impact BDNF levels by modifying the epigenetic markers of BDNF promoters. PA induces metabolic changes, and it is conceivable that an endogenous molecule induced by exercise serves as a regulator of BDNF transcription. *β*-HB, capable of crossing the BBB, accumulates in the hippocampus and upregulates BDNF expression. Furthermore, exercise triggers the activation of the sympathetic nervous system, which can have diverse effects on peripheral organs and the brain. Stimulation of β2-adrenergic receptors in rats has been shown to decrease the expression of inflammatory cytokines and increase BDNF expression in the hippocampus [43].

Astrocytes, know to play a role in regulating local blood flow in the CNS by producing and releasing various molecular mediators, display, compared with neurons, higher rates of glycolysis but lower rates of oxidative phosphorylation, indicating a preference for lactate production. Lactate serves as the primary substrate for neuronal functioning during cerebral activation and is transported between astrocytes, which act as a “lactate source,” and neurons, which act as a “lactate sink,” through astrocyte–neuron lactate shuttle (ANLS) [44]. Accumulating evidence demonstrates that PA, activating ANLS, promotes BDNF expression, supporting synaptic plasticity [45].

In a tripartite synapse, neurotransmitters released by neurons bind to receptors not only on neighboring neurons but also on adjacent astrocyte processes. This activation leads to an increase in intracellular calcium concentration, which can subsequently spread to other astrocytes. The elevated calcium levels trigger the release of gliotransmitters, including GLU, *γ*-aminobutyric acid (GABA), ATP, adenosine, *D*-serine, and BDNF. Through tripartite synapses, astrocytes play a crucial role in maintaining transmitter homeostasis, particularly for GLU [46].

FNDC5, highly abundant in the brain, gives rise to the cleaved form of irisin, which has been detected in human cerebrospinal fluid and hypothalamic regions, particularly in paraventricular neurons. The increased expression of FNDC5 mRNA in the hippocampus following exercise coincides with the expression of BDNF [47]. Consequently, the simultaneous upregulation of FNDC5 and BDNF mRNAs in hippocampal neurons after exercise suggests a potential role of irisin in neuronal survival, activity, and cognitive functions [48]. The neuroprotective properties of irisin have been demonstrated in an in vitro study, where it protected neuronal cells (PC12) from ischemic injury by activating the Akt and ERK1/2 pathways. Irisin treatment after oxygen-glucose deprivation also reduced the activation of the NOD-, LRR-, and pyrin domain-containing protein 3 (NLRP3) inflammasome in neurons, thus modulating the postischemic innate immune response [49]. While the existing evidence indicates that irisin protects brain health and metabolic rates by inhibiting apoptosis (type I cell death), its specific effects on autophagy in the brain have not been sufficiently investigated. Irisin influences brain energy metabolism by increasing glucose uptake, elevating hexokinase protein levels, and enhancing mitochondrial complex activity in astrocytes, as demonstrated in an in vivo study [50]. Moreover, irisin treatment in cultured astrocytes upregulated ATP and glucose transporter-4 expression and activated the AMPK pathway, which regulates mitochondrial pools of NAD or nicotinamide phosphoribosyltransferase (NAMPT). NAMPT, an enzyme involved in NAD production, plays a neuroprotective role by increasing or maintaining mitochondrial NAMPT and NAD levels in brain pathways following ischemia [51].

## 5. Irisin as Potential Target for PD Prevention and Treatment

PA induces changes in the SNCA methylation status and protein levels of αSyn [52]. In healthy young and old subjects, divided depending on their level of physical exercise, platelet αSyn/tau heterocomplexes were inversely related to plasma antioxidants and the level of PA [53]. There is some evidence that resistance training can reduce OS and increase the antioxidant capacity in PD patients, highlighting the capacity of structured physical exercise to improve the oxidative state in people with PD [54].

According to animal research, aerobic exercise can inhibit OS and can improve mitochondrial function. In particular, exercise on a treadmill can increase the level of BDNF in the striatum in rat PD models. Moreover, treadmill exercise can increase the levels of Nrf2. Furthermore, long-term treadmill training could decrease the loss of dopaminergic cells and transmission and reduce the oxidation of proteins caused by neurotoxins in PD animal models [6].

A significant number of studies have shown that PA can play a role in reducing the incidence of PD. At the same time, PA can improve motor and nonmotor symptoms of PD. These effects are mediated by the reduction of αSyn protein, the reduction of OS, the upregulation of BDNF, and, particularly, the enhancement of mitochondrial function [5].

In an in vivo study, Dutta and colleagues investigated the effect of treadmill exercise in A53T αSyn model rats. Remarkably, treadmill exercise inhibited αSyn spreading in substantia nigra, reduced αSyn aggregation, and upregulated PPARα in A53T mice brain [55].

The αSyn preformed fibril (PFF) mouse model is commonly used to study sporadic PD. In this model, intravenous administration of irisin via viral vectors after the injection of αSyn PFF into the striatum resulted in a reduction in the formation of pathological αSyn aggregates. Additionally, irisin prevented the loss of DA neurons and the decrease in striatal DA levels. Irisin also significantly improved motor deficits induced by αSyn PFF, as assessed by behavioral tests such as the pole test and grip strength test. Further analysis revealed that irisin enhanced the degradation of pathological αSyn through endolysosomal pathways [56].

Animal models of PD have demonstrated that exercise induces structural and functional changes in the brain. These changes include alterations in synaptogenesis, increased levels of endogenous neurotrophic factors, enhanced angiogenesis, and reduced neuroinflammation. Exercise has also shown specific effects on the nigrostriatal pathway, such as increased DA release, decreased expression of the DA transporter, preserved striatal DA levels, and the preservation or restoration of dopaminergic neurons and terminals. These targeted effects on disease pathology suggest a neuroprotective role of exercise in slowing the progression of PD. Exercise-induced physiological changes are observed regardless of PD, indicating that exercise may build resilience and better equip individuals to cope with the disease's burden, rather than exerting specific neuroprotective effects [57].

Several studies have reported an inverse association between the risk of developing PD and the amount of PA practiced throughout life. The protective effect of PA against PD appears to be more pronounced when engaging in PA during young-to-middle adulthood and in later stages of life. Some researchers suggest that individuals who engage in PA during these periods have a 40% lower risk of PD compared to those who remain inactive [58].

The intensity of PA may also influence the risk of developing PD. A longitudinal study involving 143,325 subjects conducted over a one-year period demonstrated that individuals engaging in high-intensity PA, such as cycling, aerobics, or tennis, had a 40% lower risk of developing PD compared to those who did not engage in PA or participated in low-intensity activities such as walking or dancing. PA is now recognized as a nonpharmacological approach recommended for PD patients to mitigate the detrimental effects of the disease. It is important to emphasize that PA also helps PD patients maintain their psychomotor learning abilities [59].

When it comes to motor function, there is robust evidence supporting the positive effects of PA on individuals with PD. Specifically, strength training has been shown to enhance muscle strength and walking speed in PD patients who are not severely affected by the disease. A meta-analysis conducted by Herman, Giladi, and Hausdorff revealed that treadmill-based walking training improves spatiotemporal parameters of walking, and these improvements can be mantained for up to 2 months. According to a Cochrane review, the most effective walking program involves 30 min of walking performed five times per week for a minimum of 6 weeks [60, 61].

PA may also exert a protective effect on the dopaminergic function in animal models of PD by stimulating the expression of various neurotrophic factors and promoting angiogenesis. PA leads to an increase in DA concentration and enhances the sensitivity of DA receptors. Specifically, PA helps mitigate alterations in dopaminergic neurons within the SN and contributes to the restoration of basal ganglia function involved in motor control through adaptive mechanisms of DA and GLU neurotransmission. PA not only protects neurons and mitochondria but also elevates the levels of nigrostriatal neurotrophic factors in the substantia nigra, particularly in PD mice with moderate neurodegeneration [62].

In PD rats, aerobic training sessions lasting 20–60 min, performed five days a week for four weeks, can restore the expression of glial fibrillary acidic protein (GFAP) in the dorsal striatum. This finding suggests that astrocytes may play a role in mediating the beneficial effects of PA in PD . The decrease in GFAP expression may be associated with reduced astrocyte expansion, possibly due to increased synaptic function induced by PA in the dorsal striatum. This observation further underscores the neuroprotective role of PA. In a study involving sedentary elderly rats, it was found that they had 11% fewer Purkinje cells (cerebellar efferents) and 9% smaller soma volumes of Purkinje cells compared to elderly rats that engaged in exercise. However, the exercising elderly rats exhibited a similar number of Purkinje cells as young rats (5 months of age), indicating the potential of PA to preserve neuronal health [7].

PA has several positive effects on PD by reducing chronic OS, promoting mitochondrial biogenesis, and upregulating autophagy. Exercise also stimulates the synthesis of neurotransmitters such as DA and trophic factors such as glial-derived neurotrophic factor (GDNF), IGF-1, BDNF and FGF-2. Additionally, it enhances the expression of neurotrophic factors such as BDNF and GDNF, as well as the hormone irisin, while downregulating Bax and neuroinflammatory cytokines in the hippocampus. Notably, the regulation of BDNF through physical exercise is crucial, as BDNF plays a multifunctional role in neuronal plasticity, synaptic transmission, stress resistance, neuronal differentiation and maturation, and activation of supporting molecules such as NFκB and dopamine in neurons [63].

In PD, dopaminergic degeneration and the accumulation of cytosolic αSyn are associated with impaired clearance through the autophagy-lysosome pathway. Voluntary exercise using a running wheel was able to increase concentrations of DJ-1, Hsp70, and BDNF, while reducing *α*-synuclein aggregation in the brains of exercising mice compared to control mice. Biochemical analysis conducted in the same study demonstrated significantly higher concentrations of Hsp70, BDNF, and DJ-1 in the running mice [64].

PA elicits significant changes in hormone concentrations, including vasopressin, cortisol, *β*-endorphin, adrenocorticotropic hormone, and others, from resting levels. The neuroendocrine response is directly proportional to the exercise volume, encompassing intensity and duration. PA initiates a coordinated series of physiological responses involving the hypothalamic-pituitary-adrenal axis and sympathetic nervous system activation. Exercise modulates the release of DA, noradrenaline (NA), and serotonin (5-HT), the three main monoamine neurotransmitters, with increased levels observed during exercise [65].

Exercise training influences the extracellular levels of neurotransmitters such as DA, NA, 5-HT, GABA, and GLU. Upregulation of DA in the brain is associated with exercise-induced elevation of serum calcium, which crosses the BBB and activates the tyrosine hydroxylase enzyme involved in calcium/calmodulin-dependent DA synthesis. Moreover, exercise enhances the binding affinity between DA and its receptors. Additionally, exercise triggers neuronal adaptations in response to uncontrollable stress. The protective mechanism of PA against stress involves the expression of galanin in the locus coeruleus, which hyperpolarizes noradrenergic neurons, inhibiting their firing and suppressing NA release. NA, which targets the amygdala and frontal cortex, mitigates anxiety-related behavior when released in lower amounts [66].

In animal models of PD, exercise induces neuroprotective effects by promoting the expression of brain neurotrophic factors such as BDNF and GDNF. Regular physical exercise leads to reduced CpG methylation in the promoter IV of the BDNF gene in rats. Free-wheel running may also improve histone H3 phosphorylation/acetylation and c-Fos induction in dentate granule neurons. Furthermore, in PD mouse models, a 6-week treadmill training exercise activated a protective Nrf2-ARE (antioxidant response element)-dependent signaling pathway in the nigrostriatal region, which helped prevent the development of parkinsonism [67].

Aerobic exercise for 4 weeks has shown long-lasting improvements in both motor and nonmotor functions of PD patients. One notable outcome of this study was an increase in BDNF signaling through the TrkB receptor in the lymphocytes of patients [68].

DA plays a role in regulating emotional and reward-related brain functions, and the positive effects of PA may be possibly attributed to its ability to increase DA concentration. Notably, exercise increases the concentration of neurotransmitters, including DA, which are also activated by certain drugs and alcohol, potentially explaining the mood-enhancing effects of exercise in humans. Voluntary exercise is genetically controlled and influenced by various neuromodulators, including DA. A positive feedback system may exist, where increased DA production leads to a greater propensity for active living, resulting in further release of DA in this self-sustaining circuit [69].

In addition to medication, PA might represent a potential intervention for PD with promising effects, including reduced decline in postural and gait instability, improved overall mobility, better cognitive performance in tasks involving processing speed and cognitive control, and slower deterioration in daily activities. Neuroimaging studies suggest that the clinical benefits of exercise in PD may be associated with its positive effects on the brain structure and function [70].

As observed by De Laat and coworkers, the neuromodulatory and neuroprotective effect of PA in PD patients can reverse the expected decrease in neuromelanin concentration in the substantia nigra into a significant increase, which can be highlighted by DAT imaging using specific PET [71]. According to these results, the benefit of PA in PD patients could be better understood in the future, thanks to specific in vivo biomarker.

## 6. Conclusions and Future Directions

OS is a key element in the pathophysiological processes of diseases of the CNS, both acute and chronic degenerative. Ferroptosis, recently described, is a mechanism mediating OS-induced damage, with growing evidence of specific involvement in PD. Regular PA, properly dosed, can be a protective factor against neurodegenerative diseases. In addition, PA improves motor and cognitive performance in patients with PD.

Among the biological mediators of PA, irisin has been identified as an agent capable of inducing systemic changes and crossing the BBB. In particular, irisin may favor cytoprotective and compensatory mechanisms against OS damage mediated by ferroptosis. The most recent findings in the literature show that irisin may be able to act on key pathophysiological mechanisms of pathological αSyn deposition in PD.

Based on these data, we conclude that PA is the most important protective factor against PD in the context of lifestyle. With the increasing understanding of the mechanisms by which PA generates these beneficial effects, it will be possible to identify specific prevention and symptomatic treatment profiles for patients at risk of or already affected by PD.

## Data Availability

As a narrative review, the sources of the manuscript are represented by the bibliography list.
